# A multi-component parallel-plate flow chamber system for studying the effect of exercise-induced wall shear stress on endothelial cells

**DOI:** 10.1186/s12938-016-0273-z

**Published:** 2016-12-28

**Authors:** Yan-Xia Wang, Cheng Xiang, Bo Liu, Yong Zhu, Yong Luan, Shu-Tian Liu, Kai-Rong Qin

**Affiliations:** 1 State Key Laboratory of Structural Analysis for Industrial Equipment, Department of Engineering Mechanics, Dalian University of Technology, Dalian, China; 2 Department of Biomedical Engineering, Dalian University of Technology, Dalian, China; 3 Department of Electrical and Computer Engineering, National University of Singapore, Singapore, Singapore; 4 Department of Anesthesiology, The First Affiliated Hospital of Dalian Medical University, Dalian, China

**Keywords:** Exercise training, Wall shear stress, Parallel-plate flow chamber, Actin microfilaments, Nitric oxide

## Abstract

**Background:**

In vivo studies have demonstrated that reasonable exercise training can improve endothelial function. To confirm the key role of wall shear stress induced by exercise on endothelial cells, and to understand how wall shear stress affects the structure and the function of endothelial cells, it is crucial to design and fabricate an in vitro multi-component parallel-plate flow chamber system which can closely replicate exercise-induced wall shear stress waveforms in artery.

**Methods:**

The in vivo wall shear stress waveforms from the common carotid artery of a healthy volunteer in resting and immediately after 30 min acute aerobic cycling exercise were first calculated by measuring the inner diameter and the center-line blood flow velocity with a color Doppler ultrasound. According to the above in vivo wall shear stress waveforms, we designed and fabricated a parallel-plate flow chamber system with appropriate components based on a lumped parameter hemodynamics model. To validate the feasibility of this system, human umbilical vein endothelial cells (HUVECs) line were cultured within the parallel-plate flow chamber under abovementioned two types of wall shear stress waveforms and the intracellular actin microfilaments and nitric oxide (NO) production level were evaluated using fluorescence microscope.

**Results:**

Our results show that the trends of resting and exercise-induced wall shear stress waveforms, especially the maximal, minimal and mean wall shear stress as well as oscillatory shear index, generated by the parallel-plate flow chamber system are similar to those acquired from the common carotid artery. In addition, the cellular experiments demonstrate that the actin microfilaments and the production of NO within cells exposed to the two different wall shear stress waveforms exhibit different dynamic behaviors; there are larger numbers of actin microfilaments and higher level NO in cells exposed in exercise-induced wall shear stress condition than resting wall shear stress condition.

**Conclusion:**

The parallel-plate flow chamber system can well reproduce wall shear stress waveforms acquired from the common carotid artery in resting and immediately after exercise states. Furthermore, it can be used for studying the endothelial cells responses under resting and exercise-induced wall shear stress environments in vitro.

## Background

Endothelial cells (ECs) lining the innermost layer of vascular wall are constantly exposed to frictional pulsatile wall shear stress induced by flowing blood in the vasculature. Many in vivo and in vitro studies have demonstrated that ECs are able to recognize different wall shear stress frequencies, amplitudes and patterns through mechanosensors (e.g., receptor tyrosine kinases, G protein-coupled receptor, integrins, and glycocalyx) located in the cell membrane [1], and transduce these signals into intracellular via different signaling pathways to regulate cell structures and functions [1], and then affect vascular tone, permeability, and ECs proliferation, apoptosis, as well as the secretion of vasoactive substances [2].

Under steady and pulsatile laminar wall shear stress conditions in vitro, ECs elongate and align parallel to the blood flow direction through cytoskeletal remodeling [3]. As wall shear stress moderately increases in a certain range, ECs produce more vasodilators nitric oxide [4], prostacyclin (PGI_2_) [5], and less vasoconstrictors endothelin-1 (ET-1) [6], which are anti-inflammatory and anti-atherosclerotic. However, under oscillatory wall shear stress with high level retrograde component, ECs are polygonal, orient randomly [7] and display functions which are opposite to those discussed above under steady and pulsatile laminar wall shear stress conditions [8]. Accordingly, the cells in oscillatory wall shear stress conditions are more likely to appear endothelial dysfunction, which is believed to be the initial step of atherosclerosis formation and development [9].

Sedentary lifestyle is a cause of endothelial dysfunction and cardiovascular diseases [10], predominantly due to the decrease of wall shear stress in vessel. In contrast, reasonable exercise trainings can improve endothelial function through enhancing the expression of anti-atherogenic factors (e.g., NO, PGI_2_) [11], and decreasing atherogenic factors (e.g., ET-1, ROS) [11]. It is commonly believed that the modest increase of pulsatile wall shear stress frequencies and amplitudes leads to the alterations of abovementioned vasoactive substances concentration and then enhance endothelial function [12, 13]. In addition, it is interesting to note that exercise training can increase retrograde wall shear stress; however, its effect on endothelial cells is still elusive. Ku et al. [14] demonstrated that retrograde wall shear stress is detrimental to endothelial function; however, Green et al. [15] showed that wall shear stress with anterograde and retrograde components is a potent stimulus to promote the generation of NO. Therefore, more investigations are needed to clear the cloud on this critical issue. Since exercise training can also elevate vessel pressure and cyclic stretch, which can also affect endothelial function, it is necessary to isolate the wall shear stress from other factors resulting from exercise. Considering that this cannot be achieved in in vivo experiments, to exclude pressure, cyclic stretch and other factors, such as nervous regulation, body temperatures affected by exercise training, and to determine the key role of wall shear stress profile on endothelial function, it is necessary to simulate exercise-induced pulsatile wall shear stress of an artery, and stimulate endothelial cells with this wall shear stress alone, and then observe the cells biological responses in vitro.

Parallel-plate flow chambers [16], cone-plate chambers [17] and microfluidic chambers [18] are common platforms to create wall shear stress environments in vitro, all of which can be used to observe cellular dynamics such as morphology [19], arrangement [19] and ROS concentration [18] in real time. However, different from microfluidic chambers, large numbers of cells in other two flow chambers can be harvested after flow experiments for subsequent experiments such as RT-PCR [20], western blot [20] and immunohistochemistry [21]. Using the aforementioned chambers, different wall shear stress waveforms were generated [17, 22]. For example, Helmlinger simulated pulsatile wall shear stress using the parallel-plate flow chamber with a glass syringe [23]. Blackman et al. [17] developed a multi-component cone-plate chamber to mimic abdominal aorta and brachial artery wall shear stress waveforms respectively. Nevertheless, the above wall shear stress waveforms were in resting state. In recent years, researchers have realized that wall shear stress is a key factor in mediating exercise-induced endothelial function variations. Estrada et al. [22] mimicked exercise-induced wall shear stress waveform using an endothelial cells culture model, but this waveform was different from the physiological condition waveform due to lack of retrograde flow. Chin et al. [18] utilized a pulsation free pump to generate exhaustive exercise-induced wall shear stress condition in the microfluidic chip. Despite convenience, microfluidic chip can be hardly utilized repeatedly. Moreover, the pulsation pump is costly [18], and it is difficult to harvest cells in the microfluidic chip for the ensuing experiments as stated previously.

In the present study, based upon a lumped parameter hemodynamics model, we have designed and fabricated a system with a reusable parallel-plate flow chamber, as well as other inexpensive and convenient adjustable peripheral components. Through carefully adjusting the system components according to the model parameter values from numerical simulations, both resting and exercise-induced wall shear stress waveforms acquired from the common carotid artery of a healthy volunteer can be replicated. To test the effectiveness of this system, we cultured HUVECs within the parallel-plate flow chamber and investigated the changes of actin microfilament and NO production level under resting and exercise-induced wall shear stress waveforms.

## Methods

### The acquisition of in vivo wall shear stress waveforms at resting and immediately after exercise states

In order to replicate resting and exercise-induced wall shear stress waveforms in vitro, the in vivo wall shear stress waveforms at the above two physiology conditions in the common carotid artery of a healthy volunteer were acquired first. Before and immediately after exercise training by acute aerobic cycling for 30 min, the inner diameter and the center-line blood flow velocity of the right common carotid artery were measured using a color Doppler ultrasound (ProSound Alpha 7, Aloka). The heart rate, brachial systolic and diastolic pressures were synchronously measured with an electronic sphygmomanometer (Patient Monitor PM8000, Mindray). According to above five variables, the in vivo wall shear stress characteristic values, including maximal wall shear stress (*τ*_*w*−max_), minimal wall shear stress (*τ*_*w*−min_), mean wall shear stress (*τ*_*w*−*mean*_), oscillatory shear index (OSI), and wall shear stress waveforms were calculated through the computational method described by Liu et al. [24]. The present study was approved by the Ethics Committee, Dalian University of Technology, China. The subject provided written informed consent before measurement.

### Multi-component parallel-plate flow chamber system

**Fig. 1:**
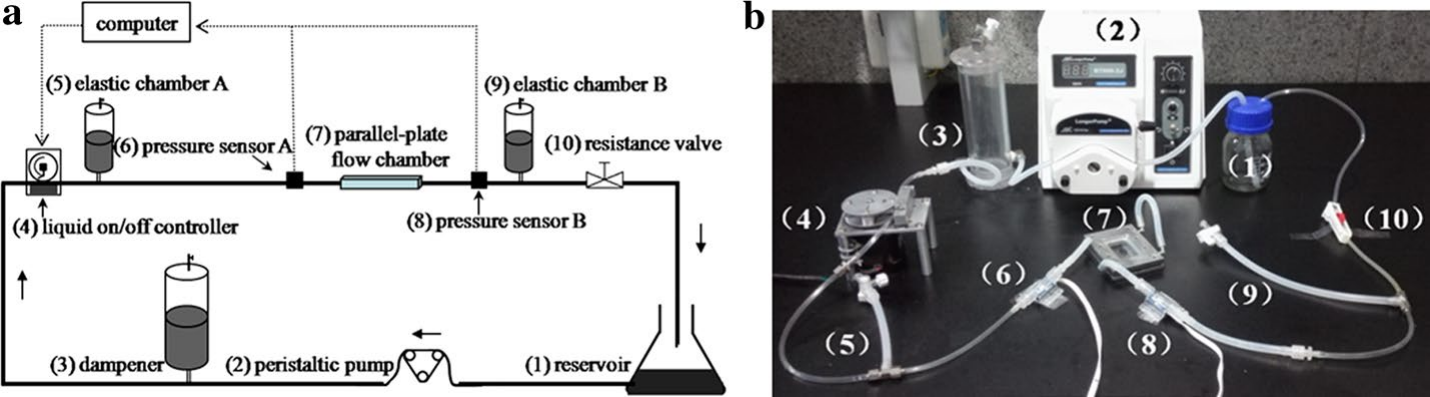
Schematic diagram (**a**) and actual diagram (**b**) of the parallel-plate flow chamber system. *1* reservoir *2* peristaltic pump *3* dampener *4* liquid on/off controller *5* elastic chamber A *6* pressure sensor A *7* parallel-plate flow chamber *8* pressure sensor B *9* elastic chamber B *10* resistance valve

The parallel-plate flow chamber system (see Fig. 1a, b) consisted of three parts: (i) a parallel-plate flow chamber; (ii) peripheral assisting components; (iii) measurement components. The structure of the parallel-plate flow chamber designed for the present study was similar to that described by Galbraith et al. [3]. The inner of the flow chamber was a 50 mm length × 12 mm width × 0.5 mm height rectangular channel. The peripheral assisting components consisted of reservoir, peristaltic pump, dampener, liquid on/off controller, elastic chamber A, elastic chamber B, and resistance valve. The reservoir was a glass media bottle with two holes on the bottle cap used as the inlet and outlet of the silicone tubes. The peristaltic pump (BT600-2J Longerpump, China) was purchased from a commercial company. The dampener, the liquid on/off controller, the pressure sensors, and the parallel-plate flow chamber were self-designed. The elastic chambers A and B were two sections of silicone tubes (inner diameter, 3.1 mm) sealed air columns in them, and the resistance valve was a flow regulator originally used for drug injection.

The compliances of the elastic chambers were calculated using the following equation:

1 $$C = \frac{dV}{dP} = \frac{Ah}{{\;n \times (P_{0} + P_{a} )}}$$

where *V* is the air volume in the elastic chamber, and *P* is the air pressure in the elastic chamber. *n* is a polytropic exponent (n ≥ 1), the process of regulating compliance is under constant temperature, so *n* = 1. *A* is the inner diameter of elastic chamber, *h* is the height of air column, *P*_0_ and *P*_*a*_ are atmosphere pressure and hydraulic pressure acting on air column in the elastic chamber, respectively. Therefore, the *h* can be obtained when the desired compliance *C* is known.

The liquid inductance of silicone tube in the flow loop was calculated as:

2 $$L = \frac{{\rho l^{\prime }}}{{A^{\prime }}}$$

where *ρ* is the fluid density, *l* ^′^ is the length of silicone tube, and *A* ^′^ is the inner diameter of the silicone tube in the flow loop.

The measurement components contained two pressure sensors (A and B) and a computer. The pressure difference of the sensor A and the sensor B was equal to the pressure drop in the flow chamber, which could be displayed in real time on the computer.

### Adjustment of the parallel-plate flow chamber system to replicate in vivo wall shear stress waveforms

**Fig. 2:**
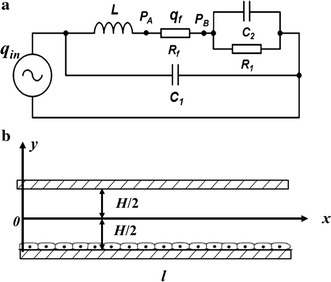
**a** Lumped parameter model for global hemodynamics of the parallel-plate flow chamber system. *q*_*in*_ the input flow rate of the total system; *q*_*f*_ the flow rate through the parallel-plate flow chamber; *P*_*A*_ and *P*_*B*_ the pressures at the two ends of the parallel-plate flow chamber, respectively; *C*_*1*_ the compliance of the elastic A; *C*_*2*_ the compliance of the elastic B; *L* the liquid inductance of silicone tube in the flow loop; *R*_*1*_ the resistance of the resistance valve; *R*_*f*_ the flow resistance of the parallel-plate flow chamber. **b** Schematic diagram of the parallel-plate flow chamber

In order to set reasonable parameter values of the system components to replicate the in vivo resting and exercise-induced wall shear stress waveforms, the global hemodynamics of the parallel-plate flow chamber system as shown in Fig. 1a was simplified as a lumped parameter model as shown in Fig. 2a, of which the governing equations are as follows:

3 $$\left\{ {\begin{array}{*{20}l} {q_{f} = \frac{{P_{A} - P_{B} }}{{R_{f} }} = C_{2} \frac{{dP_{B} }}{{dt}} + \frac{{P_{B} }}{{R_{1} }}} \\ {q_{{in}} - q_{f} = C_{1} \frac{{d\left( {P_{A} + L\frac{{dq_{f} }}{{dt}}} \right)}}{{dt}}} \\ \end{array} } \right.$$

where, *q*_*in*_ is the input flow rate of the total system; *q*_*f*_ is the flow rate through the parallel-plate flow chamber; *P*_*A*_ and *P*_*B*_ are the pressures at the two ends of the parallel-plate flow chamber, respectively; *C*_*1*_ and *C*_*2*_ are the compliances of the elastic A and B, respectively; *L* is the liquid inductance of silicone tube in the flow loop; *R*_*1*_ is the resistance of resistance valve; *R*_*f*_ is the flow resistance of the parallel-plate flow chamber. Numerical simulations demonstrated that the waveform of pulsatile flow rate *q*_*f*_ through the parallel-plate flow chamber could have anterograde and retrograde components (data shown in the “Results” section) by setting the appropriate values for *L*, *C*_*1*_ and *C*_*2*_, and satisfying with *C*_*2*_ > *C*_*1*_ in the lumped parameter model.

The local hemodynamics in the parallel-plate flow chamber as shown in Fig. 2b was described by simplified Navier–Stokes equation as follows:

4 $$\frac{\partial u}{\partial t} = - \frac{1}{\rho }\frac{\partial p}{\partial x} + \frac{\eta }{\rho }\frac{{\partial^{2} u}}{{\partial y^{2} }}$$

where, *u* = *u*(*y*, *t*) is the fluid velocity along *x*-direction, *p* = *p*(*x*, *t*) is the pressure, *t* is the time, *η* is the fluid viscosity, *ρ* is the fluid density. Under pulsatile pressure gradient, $\frac{\partial p}{\partial x}$, the solution of Eq. (4) is expressed as:

5 $$u(y,t) = \sum\limits_{n = - \infty }^{ + \infty } {\left[ {\frac{{ch\left( {\frac{y}{H}\alpha_{n} j^{{\frac{1}{2}}} } \right)}}{{ch\left( {\frac{1}{2}\alpha_{n} j^{{\frac{1}{2}}} } \right)}} - 1} \right]} \frac{{A\left( {\omega_{n} } \right)}}{{j\rho \omega_{n} }}e^{{j\omega_{n} t}}$$

In the formula, *y* is the coordinate along height direction, *H* is the height of the flow chamber, $\alpha_{\text{n}} = H\left( {\frac{{\rho \omega_{\text{n}} }}{\eta }} \right)^{{\frac{1}{2}}}$ is the Womersley number corresponding to *n*-th harmonic component,$j = \sqrt { - 1}$, *A*(*ω*_*n*_) as the *n*-th harmonic component of the pressure gradient corresponding to the circular frequency *ω*_*n*_. The flow rate *q*_*f*_ (t) can be expressed as:

6 $$q_{f} (t) = WH\sum\limits_{n = - \infty }^{ + \infty } {\left[ {\frac{2}{{\alpha_{n} j^{{\frac{1}{2}}} }}th\left( {\frac{1}{2}\alpha_{n} j^{{\frac{1}{2}}} } \right) - 1} \right]} \frac{{A\left( {\omega_{n} } \right)}}{{j\rho \omega_{n} }}e^{{j\omega_{n} t}}$$

where, *W* is the width of the flow chamber.

From Eqs. (5 and 6), the fluid velocity *u*(*y*, *t*)can be expressed by the harmonic component *q*_*fn*_(*ω*_*n*_) of flow rate *q*_*f*_(*t*) as,

7 $$u(y,t) = \frac{1}{WH}\sum\limits_{n = - \infty }^{ + \infty } {\frac{{ch\left( {\frac{y}{H}\alpha_{n} j^{{\frac{1}{2}}} } \right) - ch\left( {\frac{1}{2}\alpha_{n} j^{{\frac{1}{2}}} } \right)}}{{\frac{2}{{\alpha_{n} j^{{\frac{1}{2}}} }}sh\left( {\frac{1}{2}\alpha_{n} j^{{\frac{1}{2}}} } \right) - ch\left( {\frac{1}{2}\alpha_{n} j^{{\frac{1}{2}}} } \right)}}} q_{fn} \left( {\omega_{n} } \right)e^{{j\omega_{n} t}}$$

Thus, the wall shear stress *τ*_*w*_(*t*) on the bottom of the flow chamber is expressed as:

8 $$\tau_{w} (t) = \eta \left. {\frac{{\partial {\text{u}}}}{\partial y}} \right|_{{y = - {H \mathord{\left/ {\vphantom {H 2}} \right. \kern-0pt} 2}}} = \frac{\eta }{{WH^{2} }}\sum\limits_{n = - \infty }^{ + \infty } {\frac{{j\alpha_{{_{n} }}^{2} th\left( {\frac{1}{2}\alpha_{n} j^{{\frac{1}{2}}} } \right)}}{{\alpha_{n} j^{{\frac{1}{2}}} - 2th\left( {\frac{1}{2}\alpha_{n} j^{{\frac{1}{2}}} } \right)}}} q_{fn} \left( {\omega_{n} } \right)e^{{j\omega_{n} t}}$$

In practice, the Reynolds number and the Womersley number satisfy that Re ≪ 1 and *α* ≪ 1, the quasi-steady flow assumption is valid, the fluid velocity, the flow rate and the wall shear stress can be simplified as [25, 26]:

9 $$u(y,t) = \frac{3}{2WH}\left[ {1 - \left( {\frac{2y}{H}} \right)^{2} } \right]q_{f} (t)$$

10 $$q_{f} (t) = \frac{{WH^{3} }}{12\eta }\left( { - \frac{dp}{dx}} \right) = - \frac{{WH^{3} }}{12\eta } \cdot \frac{\Delta P}{l}$$

11 $$\tau_{w} (t) = \frac{{6\eta q_{f} (t)}}{{WH^{2} }} = - \frac{H}{2} \cdot \frac{dp}{dx} = \frac{H}{2} \cdot \frac{\Delta P}{l}$$

here, $\frac{dp}{dx}$ can be determined from the measurement of the pressure drop, *ΔP*, divided by the length of the flow chamber, *l.* During the experiments, *ΔP* was acquired through pressure sensors A (*P*_*A*_) and B (*P*_*B*_) (*ΔP* = *P*_*A*_ − *P*_*B*_) in real time. Since *H* and *l* are known, so wall shear stress *τ*_*w*_(*t*) can be determined. The flow resistance of the parallel-plate flow chamber can be expressed as:

12 $$R_{f} = \frac{\Delta P}{{q_{f} (t)}} = \frac{12\eta l}{{WH^{3} }}$$

The degree of flow reversal is quantified by OSI, which was calculated using the following definition [14]:

13 $$OSI = \frac{1}{2}\left. {\left( {1 - \frac{{\left| {\int_{0}^{T} {\tau_{w} } dt} \right|}}{{\int_{0}^{T} {\left| {\tau_{w} } \right|dt} }}} \right.} \right)$$

where *T* is a cardiac cycle.

Given the desired wall shear stress *τ*_*w*_(*t*) in the parallel-plate flow chamber, the flow rate *q*_*f*_(*t*) through the flow chamber can be calculated by Eq. (11). To fit this flow rate *q*_*f*_(*t*), the values of parameters for the parallel-plate flow chamber and the fluid adopted in the numerical simulations were listed in Table 1, the values for all the global hemodynamic parameters in the lumped parameter model, including the compliances *C*_*1*_ for the elastic chamber A, *C*_*2*_ for the elastic chamber B, the inductance of flow loop tube, the inductance *L*, as well as the resistance, *R*_*1*_, were determined by trial and error method with Matlab/Simulink software (The Math Works R2010a, Inc.) as shown in Table 2.

**Table 1:** Values of parameters for the parallel-plate flow chamber and the fluid

Parameters	Values
*l*	5 cm
*W*	1.2 cm
*H*	0.05 cm
*η*	0.001 Pa.s

**Table 2:** Values of the global hemodynamic parameters in the lumped parameter model

	*L*/pa s^2^ ml^−1^	*R*_*1*_/pa s ml^−1^	*R*_*f*_/pa s ml^−1^	*C*_*1*_/ml pa^−1^	*C*_*2*_/m pa^−1^
Resting	50	1570	400	3.50 × 10^−5^	3.85 × 10^−5^
Immediately after exercise	39	1189	400	4.63 × 10^−5^	5.09 × 10^−5^

Based on the values for the global hemodynamic parameters in the lumped parameter model (Table 2), the rotating rate of peristaltic pump, the frequency of liquid on/off controller, as well as the values of the elastic chambers (A and B) and the resistance valve could be adjusted to generate the resting and exercise-induced wall shear stress waveforms, respectively.

### Cells culture

Human umbilical vein endothelial cells (HUVECs) line were derived from Dalian Medical University, and cultured in Dulbecco’s modified Eagle’s medium/Ham’s nutrient mixture F12 (DMEM/F12 1:1, Gibco, USA) supplement with 10% (v/v) fetal bovine serum (FBS, Hyclone, USA), 1% (v/v) penicillin/streptomycin (PS, Gibco, USA) and 1% (v/v) l-glutamine (Solarbio, China) at 37 °C with 5% CO_2_ and 95% air. Confluent HUVECs in 60 mm petri dish were trypsinized with 1 ml 0.25% trypsin and 0.05% EDTA (Hyclone, USA) for 5 min at 37 °C, and terminated by 1 ml complete culture medium. The collected cells suspension solution were centrifuged and re-suspended in complete culture medium, and then were seeded on 3 μg/cm^2^ collagen-coated (Solarbio, China) coverslips with the density of 3 × 10^5^ cells/ml. When cells were confluence over 80%, the FBS in culture media was changed into 0.5% in preparation for the following flow perfusion.

### Flow perfusion experiments

Prior to the flow perfusion, one coverslip with cultured cells was randomly selected as static control group, which would be remain still in cell incubator without a fluidic flow, and two other coverslips chosen to as the experiment groups would be subjected to either resting or exercise-induced wall shear stress waveform. The flow chamber assembled with coverslip was connected to parallel-plate flow chamber system in a super-clean worktable, and this system was put into the cells incubator and running in it during the experiments. The cells of experimental groups were exposed to resting and exercise-induced wall shear stress waveforms for 6 h. After 6 h, the coverslips with cells were taken out for the further analysis. Each experiment was repeated at least three times.

### Fluorescence staining of the actin microfilaments

Actin microfilaments were visualised using FITC labeled Phalloidin (YEASEN, China). After flow perfusion experiments, the cells were rinsed twice with 37 °C warm 1× PBS (Solarbio, China) firstly. Then, the cells were fixed with 4% formaldehyde in 1× PBS at room temperature for 10 min, permeabilized with 0.5% Triton-X-100 (Beyotime, China) for 5 min, and incubated by 200 μl 100 nM FITC Phalloidin at room temperature out of direct sunlight for 30 min. After each of the above steps, the cells were washed three times with 1× PBS for 5 min each. Subsequently, the cells were imaged by inverted fluorescence microscope (Olympus, Japan) equipped with a charge–coupled device (CCD) camera using appropriate exciting light. Likewise, the same procedures were carried out for the static control groups.

### Detection of NO in the HUVECs

4-Amino-5-methylamino-2′,7′-difluorofluorescein diacetate (DAF-FM DA, Beyotime, China) was used to detect the production of intracellular NO. The fluorescent intensity of DAF-FM DA itself is very weak, but it is enhanced by interacting with NO, due to the benzotriazole generation. After stimulating with wall shear stress, the cells were immediately washed twice with 1× PBS, loaded with 15 μmol/L DAF-FM DA in the cell incubator for 30 min, and rinsed three times with 1× PBS. Intracellular NO was evaluated using fluorescence microscope (Olympus, Japan) with equipped CCD camera. The fluorescence excitation and emission wavelengths are 495 and 515 nm, respectively. Similarly, the same procedures were conducted for the static control groups.

## Results

### In vivo resting and exercise-induced wall shear stress waveforms in the common carotid artery

**Fig. 3:**
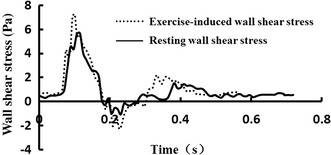
The in vivo resting and exercise-induced wall shear stress waveforms in one cardiac cycle

Figure 3 shows in vivo resting and exercise-induced wall shear stress waveforms in the common carotid artery of a healthy volunteer acquired by the color Doppler ultrasound. In contrast to resting condition, the frequency and the amplitude of exercise-induced wall shear stress waveform increases, moreover, it is worth noting that the reversing part of the wall shear stress waveform also obviously enhances. These resting and exercise-induced wall shear stress waveforms shown in Fig. 3 are the desired wall shear stress waveforms to be numerically and experimentally replicated in the parallel-plate flow chamber.

### Replication of resting and exercise-induced wall shear stress waveforms in the common carotid artery using the parallel-plate flow chamber system

**Fig. 4:**
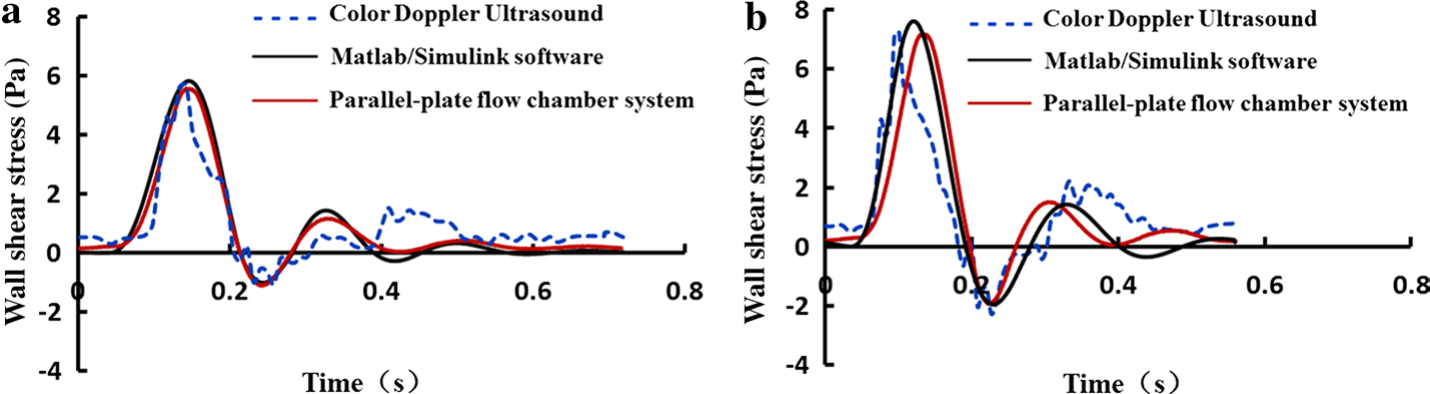
Wall shear stress waveforms acquired from the common carotid artery in vivo and separately produced by Matlab/Simulink software and experiments in the parallel-plate flow chamber system. **a** Resting wall shear stress. **b** Exercise-induced wall shear stress

Figure 4 exhibits the common carotid artery in vivo and separately produced by Matlab/Simulink software and experiments in the parallel-plate flow chamber system. It is observed from Fig. 4 that the wall shear stress waveforms from both numerical simulations and experimental measurements in the parallel-palate flow chamber are in agreement with that measured by ultrasound color Doppler in vivo. In addition, the characteristic values (*τ*_*w*−max_, *τ*_*w*−min_, *τ*_*w*−*mean*_, OSI) of the resting and exercise-induced wall shear stress waveforms numerically and experimentally reproduced in parallel-plate flow chamber system are similar to those acquired by the color Doppler ultrasound within ±10% difference in the same states (data shown in Table 3).

**Table 3:** Characteristic values of the resting and exercise-induced wall shear stress waveforms acquired by the color Doppler ultrasound from the common carotid artery of a healthy volunteer and reproduced by the parallel-plate flow chamber system

Acquisitions of wall shear stress waveforms	Forms of wall shear stress waveforms	τ_w−max_/Pa	τ_w−min_/Pa	τ_w−mean_/Pa	OSI
Color doppler ultrasound	Resting	5.67	−1.06	0.89	0.060
	Exercise-induced	7.26	−2.15	1.18	0.105
Parallel-plate flow chamber system	Resting	5.57	−1.10	0.80	0.066
	Exercise-induced	7.19	−1.95	1.16	0.095

### Effects of wall shear stress on actin microfilaments in HUVECs

**Fig. 5:**
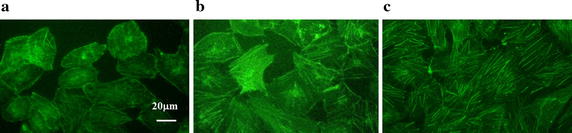
Actin microfilament responses under static condition and separately exposed in resting and exercise-induced wall shear stress waveforms for 6 h. **a** Static. **b** Resting wall shear stress. **c** Exercise-induced wall shear stress

Figure 5 exhibits that there are more actin stress fibers in cells under exercise-induced wall shear stress condition compared with those in resting wall shear stress condition; moreover, the majority of F-actin microfilaments inside cells exposed in both wall shear stress waveforms are long, continuous and paralleled with the long axis of cells, while those in static condition are short, disordered and radiated to every directions.

### Effects of wall shear stress on NO production level in HUVECs

**Fig. 6:**
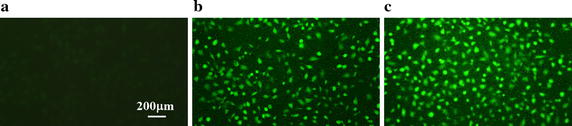
Intracellular NO level under static condition and separately exposed in resting and exercise-induced wall shear stress waveforms for 6 h. **a** Static. **b** Resting wall shear stress. **c** Exercise-induced wall shear stress

As shown in Fig. 6, HUVECs exposed in resting and exercise-induced wall shear stress both exhibit intense green fluorescence in contrast to static control group. Importantly, the fluorescent intensity of cells exposed in exercise-induced wall shear stress is stronger than those under the exposure of resting wall shear stress (see Fig. 6b, c). Since the fluorescent intensity of cells can approximately reflect NO production levels, it is concluded that cells cultured in exercise-induced wall shear stress condition produced more NO than those cultured in resting wall shear stress condition.

## Discussion

Wall shear stress imposed by the blood flow plays a vital role in mediating endothelial cells structure and function, as well as maintaining vascular endothelial homeostasis. Therefore, since early 1970s, researchers have started to create flow environments to fully understand the biological responses of cells under different wall shear stress patterns. However, those flow conditions in early investigations were mainly focused on steady [16], sinusoidal [11, 12], disturbed [27], or oscillatory flows [27]. It is not until the beginning of the twenty-first century that the pulsatile wall shear stress waveforms in arteries in vivo at resting state were reproduced in vitro [17]. In recent years, Chin et al. mimicked exhaustive exercise-induced wall shear stress profile using a microfluidic chip with pulsation free pump, and studied the effect of this wall shear stress profile on the generation of ROS [18]. It is well known that the pulsation pump is expensive, the microfluidic chips can be rarely utilized repeatedly, and the cells in the microfluidic chips are difficult to be harvested for the following molecular biology experiments. Hence, to overcome the above limitations, we designed and assembled the in vitro parallel-plate flow chamber system with a reusable parallel-plate flow chamber and other cheap and easy obtainable components, such as self-designed dampener, elastic chambers made by silicone tubes, and resistance value originally used for drug injection.

The primary characteristics of exercise-induced wall shear stress waveforms were the increases in the waveform frequency and OSI (see Fig. 3). In order to reproduce these characteristics of exercise-induced wall shear stress waveforms by the parallel-plate flow chamber system, numerical simulations with a lumped parameter model for global hemodynamics were carried out before the actual experimental system was assembled. The components of actual flow chamber system were carefully adjusted based upon the values of the global hemodynamic model parameters from numerical simulations. Both the simulation and experimental results indicated that the wall shear stress waveforms within the parallel-plate flow chamber could approximately replicate those in the carotid artery in vivo. More specifically, the maximal, minimal, mean wall shear stress as well as OSI in the parallel-plate flow chamber are similar to those in vivo acquired by the color Doppler ultrasound at resting state and immediately after exercise. However, because of the complexity of the human circulatory system, it was hard to totally reproduce the in vivo wall shear stress waveforms using the proposed multi-component parallel-plate flow chamber system.

It is notably that our system as shown in Fig. 1 could produce the wall shear stress waveforms with retrograde components which are very common in the carotid artery during exercises. The hemodynamic principle for generation of retrograde wall shear stress waveform could be explained by the lumped parameter model as shown in Fig. 2a. When the liquid controller in Fig. 1 was turned on, a part of culture media flowed into the elastic chamber A and B, and the other part of culture media flowed into the reservoir through the parallel-plate flow chamber and the resistance valve; while the liquid controller was turned off, the input flow rate was zero but the culture media in the elastic chamber A or B would continue flowing in the loop. When the compliance of elastic chamber B was larger than that of the elastic chamber A, the culture media could flow from the elastic chamber B to A through the flow chamber. Thus, the oscillatory flow with anterograde and retrograde components would occur in the flow chamber (see Fig. 4). In addition, the amplitude of the retrograde wall shear stress could be determined by the compliance ratio of the elastic chamber B to A.

To demonstrate the validity of the parallel-plate flow chamber system, we stimulated HUVECs with resting and exercise-induced wall shear stress waveforms respectively, and evaluated the responses of the actin microfilaments and NO production in endothelial cells. Actin microfilaments, as one of three types of cells cytoskeletal polymers, play important roles in keeping cells structural stability and integrity. A series of in vivo and in vitro researches have confirmed that they can response to steady [16], sinusoidal [27] and artery wall shear stress profiles [27]. In this paper, we showed that the quantity of actin microfilaments increased and mostly of them were long, continuous, and paralleled with the long axis of cells after 6 h exposure to two wall shear stress waveforms, which is consistent with the report of Galbraith et al. [3]. In addition, compared with resting wall shear stress, there were more actin microfilaments in cells subjected to exercise-induced wall shear stress waveforms mainly due to the increase of wall shear stress frequency and amplitude. At the same time, our results revealed that the change of actin microfilaments occurred earlier than the variation of cells shape and orientation under fluid flow, which is in agreement with that reported by Franke et al. [28]. NO is produced from l-arginine through the action of phosphorylation of endothelial nitric oxide synthase (p-eNOS) in vascular endothelial cells [29]. As accepted antihypertensive, antiatherosclerotic and antiaggregatory substance, NO plays crucial roles in regulating vascular tone, endothelium inflammatory reaction, platelets adhesion, as well as vascular growth and regeneration. In the present study, we detected the intracellular NO level through DAF-FM DA. The result suggested that HUVECs generated more NO exposed to exercise-induced wall shear stress than those in resting wall shear stress, which was in agreement with in vivo results [30, 31].

The responses of endothelial cells actin microfilaments and NO production level under two wall shear stress conditions confirmed the effectiveness of the parallel-plate flow chamber system which can provide effective wall shear stress profiles to cells. The system can also create other in vivo shear stress waveforms, such as disturbed flow in artery branches and curves through regulating the elements, which makes it as a great platform to carry out endothelial cells mechanobiology researches.

## Conclusion

In summary, we designed and assembled an in vitro parallel-plate flow chamber system with appropriate components based on a lumped parameter hemodynamics model. This system could well reproduce resting and exercise-induced wall shear stress waveforms acquired from the common carotid artery in vivo. The different responses of the actin microfilaments and NO production level in HUVECs under resting and exercise-induced wall shear stress waveforms also verified the feasibility of this system.

## Abbreviations

HUVECs: human umbilical vein endothelial cells
NO: nitric oxide
ECs: endothelial cells
PGI_2_: prostacyclin
ET-1: endothelin-1
ROS: reactive oxygen species
*τ*_*w*−*max*_: maximal wall shear stress
*τ*_*w*−*min*_: minimal wall shear stress
*τ*_*w*−*mean*_: mean wall shear stress
OSI: oscillatory shear index
DMEM/F12: Dulbecco’s modified Eagle’s medium/Ham’s nutrient mixture F12
FBS: fetal bovine serum
PS: penicillin/streptomycin
CCD: charge–coupled device
DAF-FM DA: 4-amino-5-methylamino-2′,7′-difluorofluorescein diacetate
p-eNOS: phosphorylation of endothelial nitric oxide synthase
